# Metabolism-associated molecular classification of *uterine corpus* endometrial carcinoma

**DOI:** 10.3389/fgene.2023.955466

**Published:** 2023-01-16

**Authors:** Munan Zhao, Wei Li

**Affiliations:** Stem Cell and Cancer Center, The First Hospital of Jilin University, Changchun, Jilin, China

**Keywords:** UCEC, metabolic classification, immunotherapy, consensus cluster, gene signature

## Abstract

*Uterine corpus* endometrial carcinoma (UCEC) is one of the most common gynecologic malignancies. Currently, for UCEC cancer, molecular classification based on metabolic gene characteristics is rarely established. Here, we describe the molecular subtype features of UCEC by classifying metabolism-related gene profiles. Therefore, integrative analysis was performed on UCEC patients from the TCGA public database. Consensus clustering of RNA expression data on 2,752 previously reported metabolic genes identified two metabolic subtypes, namely, C1 and C2 subtypes. Two metabolic subtypes for prognostic characteristics, immune infiltration, genetic alteration, and responses to immunotherapy existed with distinct differences. Then, differentially expressed genes (DEGs) among the two metabolic subtypes were also clustered into two subclusters, and the aforementioned features were similar to the metabolic subtypes, supporting that the metabolism-relevant molecular classification is reliable. The results showed that the C1 subtype has high metabolic activity, high immunogenicity, high gene mutation, and a good prognosis. The C2 subtype has some features with low metabolic activity, low immunogenicity, high copy number variation (CNV) alteration, and poor prognosis. Finally, a model was identified, with three gene metabolism-related signatures, which can predict the prognosis. These findings of this study demonstrate a new classification in UCEC based on the metabolic pattern, thereby providing valuable information for understanding UCEC’s molecular characteristics.

## Introduction

*Uterine corpus* endometrial carcinoma (UCEC) is one of the most common malignancies in the female reproductive system (Matteson et al., 2018). As predicted by Siegel et al. (2021), there were approximately 14,000 new UCEC patients in 2021 in the United States, of which 4,000 deaths occurred. Generally, due to the unstable level of estrogen, UCEC is prevalent among post-menopausal women (Chen et al., 2015). Overall, most UCEC patients can be diagnosed at an early age, with the 5-year overall survival (OS) rate reaching more than 90%. However, the prognosis of advanced or recurrent UCEC patients is also very poor, with a 5-year OS rate less than 30% (Morice et al., 2016). Many risk factors have been demonstrated to contribute to the generation and development of UCEC patients, such as smoking, drinking, overweight, and high blood pressure (Zhang et al., 2014). In particular, molecular changes are one of the factors that contribute to the development of UCEC (Li et al., 2020). Some studies have demonstrated that certain genetic alterations or molecular changes can affect UCEC patient prognosis (Bell and Ellenson., 2019). Then, in March 2020, the National Committee on Computer Network (NCCN) first recommended The Cancer Genome Atlas (TCGA) molecular subtype, indicating the era of genotype-based precision therapy has come. In the public TCGA study, endometrial cancer was divided into four subtypes, namely, POLE hyper-mutation, high mutation microsatellite instability (MSI), and non-specific molecular variation (NSMP) (Kandoth et al., 2013). Recently, despite new diagnostic methods and clinical treatments for UCEC emerging, the prognosis of UCEC patients remains very poor. Therefore, in order to develop more precise diagnoses and personalized therapies, deeply understanding the mechanisms underlying UCEC’s genetic diversity at the molecular feature level is needed. More recently, some signatures, such as immune alterations and mRNA expression pattern analyses, were utilized for molecular subtyping in many cancers. However, the relationships between the molecule features and the clinical characteristics of UCEC have not been fully studied.

Many studies have demonstrated that cancer is a metabolic-disorder disease in patients (Coller, 2014; Boroughs and DeBerardinis, 2015). In the development of cancer progression, features such as mutations and cancer-related genes will influence the metabolic procession, contain one-carbon metabolism, aerobic glycolysis, and glutaminolysis, of which all progress will support tumor cell growth and proliferation (Fiehn et al., 2016). Therefore, researching the different metabolic target genes between tumor and normal cells has become a useful therapeutic strategy. Moreover, deeply exploring molecular changes during the metabolism progress can contribute to the developmental progress of targeted therapies (Martinez-Outschoorn et al., 2017). Many studies about the metabolic subtype classification have been reported; hepatocellular carcinoma (HCC) cancer was classified into three subclasses using a panel of metabolic genes (Yang et al., 2020). In cervical cancer, based on 2,752 previously described metabolic genes, unsupervised clustering of RNA sequencing data identified three META clusters (Li et al., 2021). However, the study on the metabolism-related molecular subtype classification of UCEC has yet to be reported.

In this study, using consensus cluster analysis, UCEC RNA data from The Cancer Genome Atlas (TCGA) which was publicly available identified two metabolic subtypes based on 2,752 metabolic genes (Supplementary Table S1), namely, C1 and C2 subtypes. Then, we further investigate the prognostic characteristics, metabolic signatures, immune infiltration features, DEGs, genetic alteration, and immunotherapy responses among the two metabolic subtypes. Furthermore, using the LASSO-penalized Cox regression model, metabolism-related signatures were identified and validated.

## Materials and methods

### Data source and processing

The UCEC clinical and molecular data (including RNA expression, mutation, and CNV) were extracted from The Cancer Genome Atlas (TCGA) (https://portal.gdc.cancer.gov/) and the UCSC Xena browser (https://gdc.xenahubs.net). Normal samples and samples without key clinical features were excluded from further analyses. After filtering, 544 patients were included in the metabolic subtype analysis and training study. Of the 544 patients, 440 patients had mutation data, and 533 patients had cnv data. For validation, 544 TCGA patients were randomly divided into 7:3 (380 samples:164 samples) and were separately selected as two validation datasets. Additional processed microarray data of 91 UCEC samples from GSE17025 (based on the GPL570 platform) were used for external validation.

### Identification of UCEC subtypes

According to the previously published 2,752 metabolism-related genes encoding all known human metabolic and transport enzymes (Possemato et al., 2011), the ConsensusClusterPlus R package (Wilkerson and Hayes, 2010) was used for unsupervised decomposition and clustering, using 1,000 rounds of hc clustering, with a maximum of k = 10 clusters. The distance matrix was set to Pearson correlation (distance), and linkage function was set as ward. D (innerLinkage) and average (finalLinkag). K of clusters was identified by selecting the optimal number of clusters based on the inspection of plots, dendrograms, and features provided by the ConsensusClusterPlus output.

### Immune infiltration estimation of UCEC subtypes

First, the CIBERSORT R package (https://cibersortx.stanford.edu/) was used to evaluate the LM22 gene signatures in UCEC subtypes (Newman et al., 2019). Then, the consensus ESTIMATE (Estimation of STromal and Immune cells in MAlignant Tumor tissues using Expression) algorithm with the ESTIMATE R package was employed to measure ESTIMATE, immune and stromal scores, which reflected the immune and stromal cell gene signature enrichment (Yoshihara et al., 2013).

### Differentially expressed genes associated with UCEC subtypes and generated gene subtypes for validation

DEGs among the UCEC subtypes were identified using the R edgeR package (Robinson et al., 2010). Genes with | log2FC| > 1 and FDR <.05 were regarded as DEGs. The aforementioned DEGs were utilized for gene clustering using the ConsensusClusterPlus R package (Wilkerson and Hayes, 2010).

### Functional and pathway enrichment analysis

GO and KEGG enrichment analyses and visualization of UCEC subtypes and DEGs subtypes were performed *via* “clusterProfiler” R package (Yu et al., 2012). Gene set variation analysis (GSVA) is an unsupervised and non-parametric gene set enrichment approach that estimates biosignature scores or pathways based on transcriptomic data (Hänzelmann et al., 2013). We downloaded the gene sets from MSigDB (Broad Institute) (Subramanian et al., 2005) and chose c2. cp.kegg.v7.0. symbols.gmt, which was used to compare the differences in metabolisms between UCEC subtypes.

### Mutation and CNV differences between UCEC subtypes

The Mutation Annotation Format (MAF) files which contain the mutation information and the seg file which contains the CNV information of the UCEC training set were downloaded and processed. The “maftools” R package was used to analyze gene mutations among UCEC subtypes (Mayakonda et al., 2018). The “svpluscnv” R package (Lopez et al., 2021) and GISTIC2 software (Mermel et al., 2011) were utilized to analyze cnv segments between UCEC subtypes.

### Immunotherapy response prediction of UCEC

The tumor immune dysfunction and exclusion (TIDE) (Jiang et al., 2018), a new computing architecture that integrates the data on two tumor immune escape mechanisms, was applied to predict the potential response to immune checkpoint blockade (ICB) therapy. Here, we used the UCEC TCGA expression data to predict the differences in response to immunotherapy for each UCEC subtype and the cell types that affect T-cell infiltration in tumors, including cancer-associated fibroblasts, myeloid-derived suppressor cells, and tumor-associated M2 macrophages.

Immunogenicity is determined by a variety of immune-related genes, including genes related to effector cells, immunosuppressive cells, major histocompatibility complex molecules, and immune regulatory factors. Using machine learning, the immune-phenotyping score (IPS) can unbiasedly assess and quantify immunogenicity. To evaluate the effect of immunotherapy, we downloaded the IPS of patients with UCEC from the TCIA database (https://tcia.at/) and compared the IPS between the metabolic subtypes.

### Establishment of the metabolic risk score model

LASSO-penalized Cox regression model was built by using the “glmnet” R package (Wu et al., 2020), and the lambda.1se, a penalty parameter for preventing overfitting, was selected to construct an optimal and prognostic gene set. Finally, the risk score of each UCEC patient was calculated by the following formula: risk score = $\sum_{i=1}^{N}Exp_{i}*\beta_{i}$ .

### Gene expression verification in metabolic risk score model

mRNA and protein expression levels of genes in tumor and normal samples were obtained from the UALCAN database (http://ualcan.path.uab.edu/) (Chandrashekar et al., 2017) and The Human Protein Atlas database (https://www.proteinatlas.org/) (Uhlén et al., 2015; Uhlen et al., 2017). Furthermore, genetic alteration of genes in the model was derived from the cBioPortal database (https://www.cbioportal.org/) (Cerami et al., 2012; Gao et al., 2013).

### Statistical analysis

Survival analyses of patients with different metabolic subtypes of UCEC were performed by the Kaplan–Meier method and compared with the log-rank test. For comparisons between two UCEC subtypes, statistical significance was estimated using unpaired Student’s t-tests and Wilcoxon tests for normally distributed variables and abnormally distributed variables, respectively. The ROC curve was analyzed, and the area under the curve (AUC) was calculated using the ‘‘survivalROC’’ package. Univariate Cox regression, LASSO analysis, and multivariate regression were then used sequentially to identify genes of prognostic significance. All calculations and statistical analyses were conducted using R (version 4.0.3), and all tests were two-sided; *p* < .05 was considered statistically significant.

## Results

### Metabolic molecular subtype identification and validation in UCEC

**FIGURE 1:**
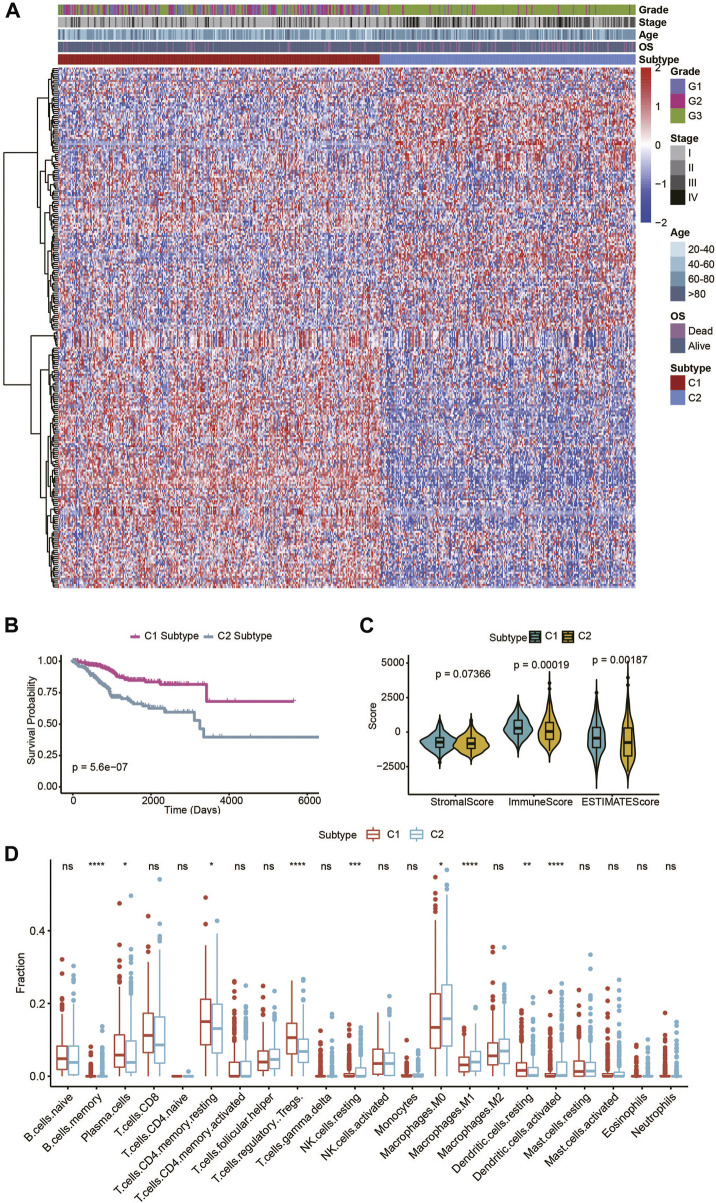
Identification of two metabolic subtypes in TCGA UCEC patients. **(A)** Consensus clustering of UCEC patients based on 255 identified metabolic genes. **(B)** OS of the two metabolic subtypes in the UCEC patients. **(C)** The violin plot of the stromal score, immune score, and ESTIMATE score of the two metabolic subtypes. **(D)** Immune cell components that differ between the two metabolic subtypes in UCEC patients.

A workflow diagram of this study is presented in Supplementary Figure S1A. For the consensus cluster analysis, 2,752 human metabolism-related genes were collected based on previous report studies (Supplementary Table S1) (Matteson et al., 2018), the mRNA expression matrix of these 2,752 metabolism-related genes in the training set was acquired from 544 TCGA UECE patients. First, genes with low expression were filtered. Then, the standard deviation (SD) for each gene was calculated, and genes with an SD value larger than 1 were selected for further analysis. After filtering, 255 genes were selected for subsequent analysis, and clustering of the UCEC patients was performed based on the aforementioned genes, using the ConsensusClusterPlus package in R. From the comprehensive clustering results, K = 2 was determined to be the best clustering number (Supplementary Figures S1B, S1C). Thus, two subtypes were identified in the UCEC training set. There were 303 patients in subtype cluster 1 (C1) and 241 patients in subtype cluster2 (C2) (Figure 1A). The survival analysis demonstrated the significant difference in patients’ OS time among the UCEC two subtypes (*p* = 5.6e-07) (Figure 1B), indicated the prognostic value in UCEC.

In order to validate the stability of molecular subtypes, we further selected GSE17025 datasets for clustering. The clustering results of molecular subtypes in GSE17025 datasets were consistent with those in TCGA, and the relevant results are shown in Supplementary Figure S2.

ESTIMATE can be used to determine the presence of stromal cells and the infiltration of immune cells in tumor samples, based on gene expression data. In this study, the ESTIMATE software was applied to estimate the stromal score, immune score, and ESTIMATE score of UCEC patients based on their transcriptional profiles. Significant differences in the ESTIMATE and immune scores, but insignificant differences in the stromal score, were presented among the two UCEC metabolic subtypes (Figure 1C). Next, we evaluated the immune infiltration landscape among UCEC metabolic subtypes using CIBERSORT software. In accordance, there were significant differences in immune cells (including B cell memory, plasma cells, T cells, CD4 memory resting, Tregs, NK cell resting, macrophage M0, macrophage M1, dendritic cells resting, and dendritic cells activated) among the two UCEC metabolic subtypes. In addition, these data illustrated that the two UCEC metabolic subtypes maintained different immune signatures (Figure 1D).

### Correlation of metabolism-related signatures and cancer pathways of UCEC metabolic subtypes

**FIGURE 2:**
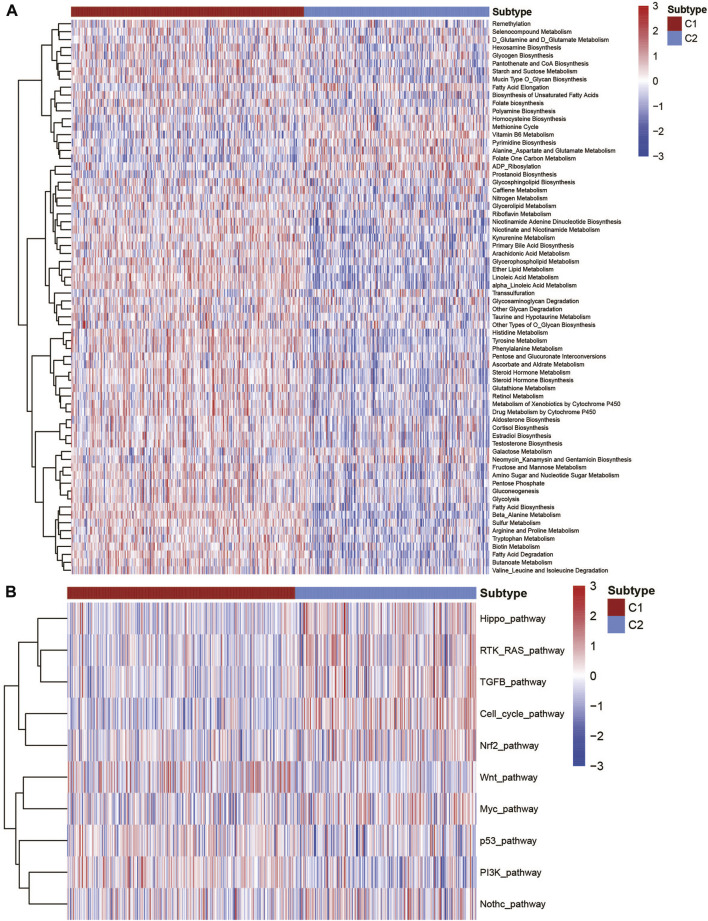
Association with the metabolism and cancer-associated pathways among UCEC metabolic subtypes. **(A)** Heatmap of the metabolic signatures of the UCEC metabolic subtype. **(B)** Heatmap of the cancer-associated signatures of the UCEC metabolic subtype.

To better describe the classification among the metabolic subtypes of UCEC patients, we further studied whether different subtypes in UCEC patients had different metabolic characteristic features. First, 115 metabolic signatures (Wang et al., 2020) were listed and quantified by the GSVA R package using the ssGSEA algorithm (Supplementary Table S2). Each patient got a score for the corresponding metabolic pathway. After the filter, 70 metabolism-associated signatures were exhibited by a heatmap (Figure 2A). It clearly showed that the C1 subtype showed active metabolism compared with the C2 subtype. Among the specific pathways, the C1 subtype was significantly associated with most glucose, lipid, and amino acid metabolic signatures, while the C2 subtype was associated with fatty acid elongation, vitamin B6 metabolism, and other metabolism signatures. Several cancer-relevant pathways were also studied (Rosario et al., 2018; Sanchez-Vega et al., 2018). The results exhibited that the C1 subtype has a significantly higher expression in the wnt pathway, p53 pathway, and PI3K pathway, while the C2 subtype has higher expression in the Hippo pathway, RTK-RAS pathway, cell cycle pathway, TGF-β pathway, and so on (Figure 2B).

Moreover, in order to understand the relationship between these metabolic signatures and immune infiltration cells, we first used the CIBERSORT algorithm to evaluate the immune infiltration of each sample. Further analysis of the correlation between metabolic signatures and immune infiltration indicated that plasma cells, Tregs, and NK-activated cells are associated with many metabolic pathways (Supplementary Figure S3). The aforementioned evidence implied that it was of high importance to explore the potential crosstalk pattern between metabolic signatures and immune infiltration cells.

### Validation performance of the UCEC metabolic subtype classification

**FIGURE 3:**
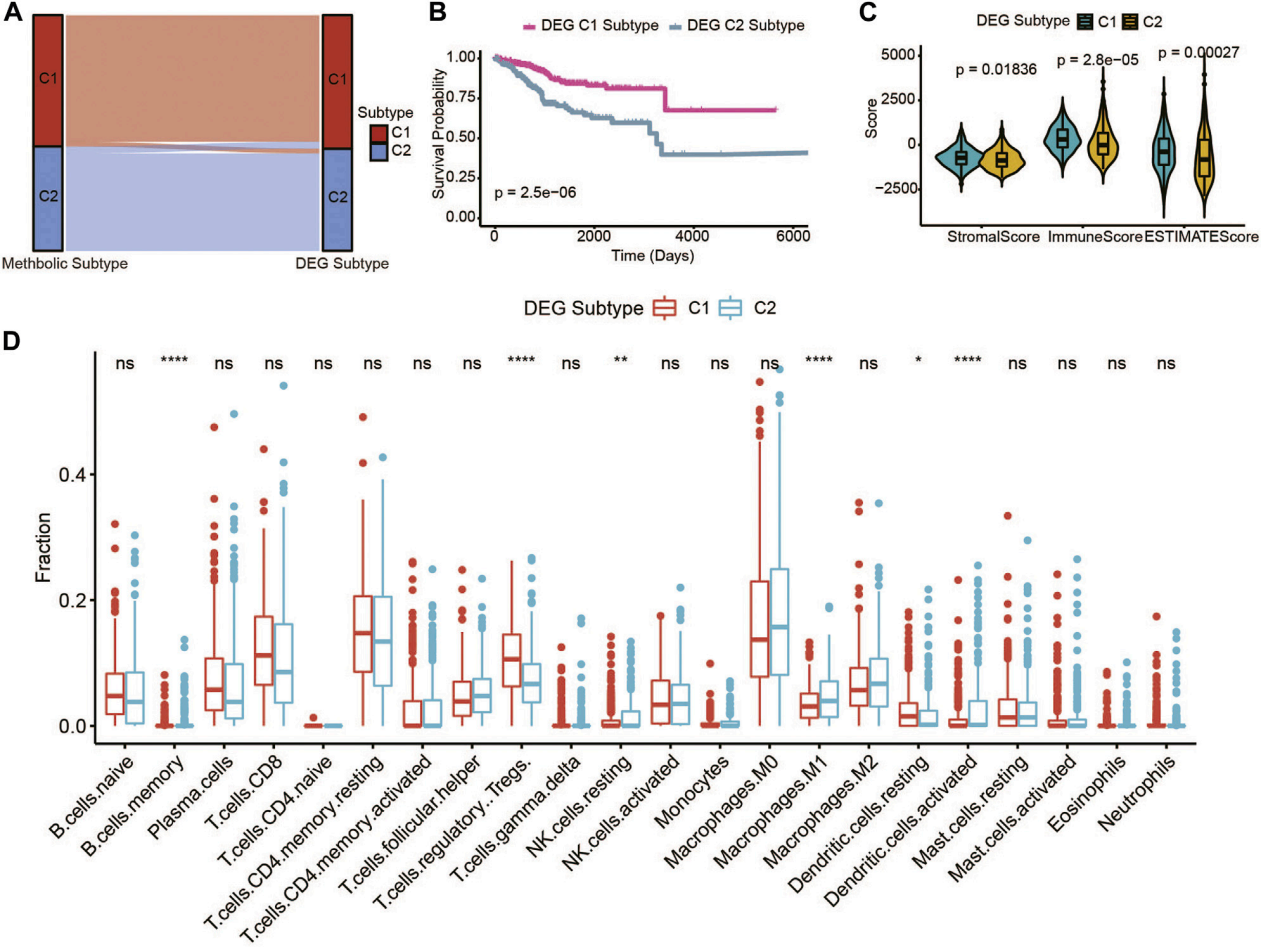
Identification of UCEC gene-clusters based on DEGs of the UCEC metabolic subtype. **(A)** Sankey diagram shows the flow change between the metabolic subtype and DEG subtype. **(B)** The OS of the two DEG gene clusters in UCEC patients. **(C)** The violin plot of stromal score, immune score, and ESTIMATE score of the two DEG gene clusters. **(D)** Immune cell components that differ between the two DEG gene clusters in UCEC patients.

To affirm the metabolic subtype of UCEC patients, an unsupervised cluster analysis of 326 of the most representative DEGs among the two metabolic subtypes obtained using the edgeR package (Robinson et al., 2010) was used to divide the UCEC patients into different subtypes (Supplementary Figure S4E). The CDF plot and consensus matrix heatmap showed that k = 2 is the optimal cluster number (Supplementary Figure S4A–D). The DEG subtype is similar with the metabolic subtype (Figure 3A). Furthermore, among these two gene subtypes, the difference in the OS was strikingly consistent with the results of the two metabolic subtypes (Figure 3B). Meanwhile, the expressions of immune and ESTIMATE scores (Figure 3C), and immune infiltration (Figure 3D), were higher in accordance with the differences among the two metabolic subtypes, which genomically verified two distinct metabolism-associated patterns in UCEC patients.

### Functional enrichment analysis of metabolic subtypes in UCEC patients

GO and KEGG analyses were used to explore the different potential molecular mechanisms and biological functions of the 326 identified DEGs among the two metabolic subtypes. The C1 subtype was associated with some transport pathways, such as transmembrane transport, potassium ion transport, potassium ion transmembrane transport, and sodium ion transport. The C2 subtype was related to processes including drug metabolism–cytochrome P450, Tyrosine metabolism, glycolysis/gluconeogenesis progress, and protein glycosylation (Supplementary Figure S5).

### Sensitivity of immunotherapy among the metabolic subtypes of UCEC patients

**FIGURE 4:**
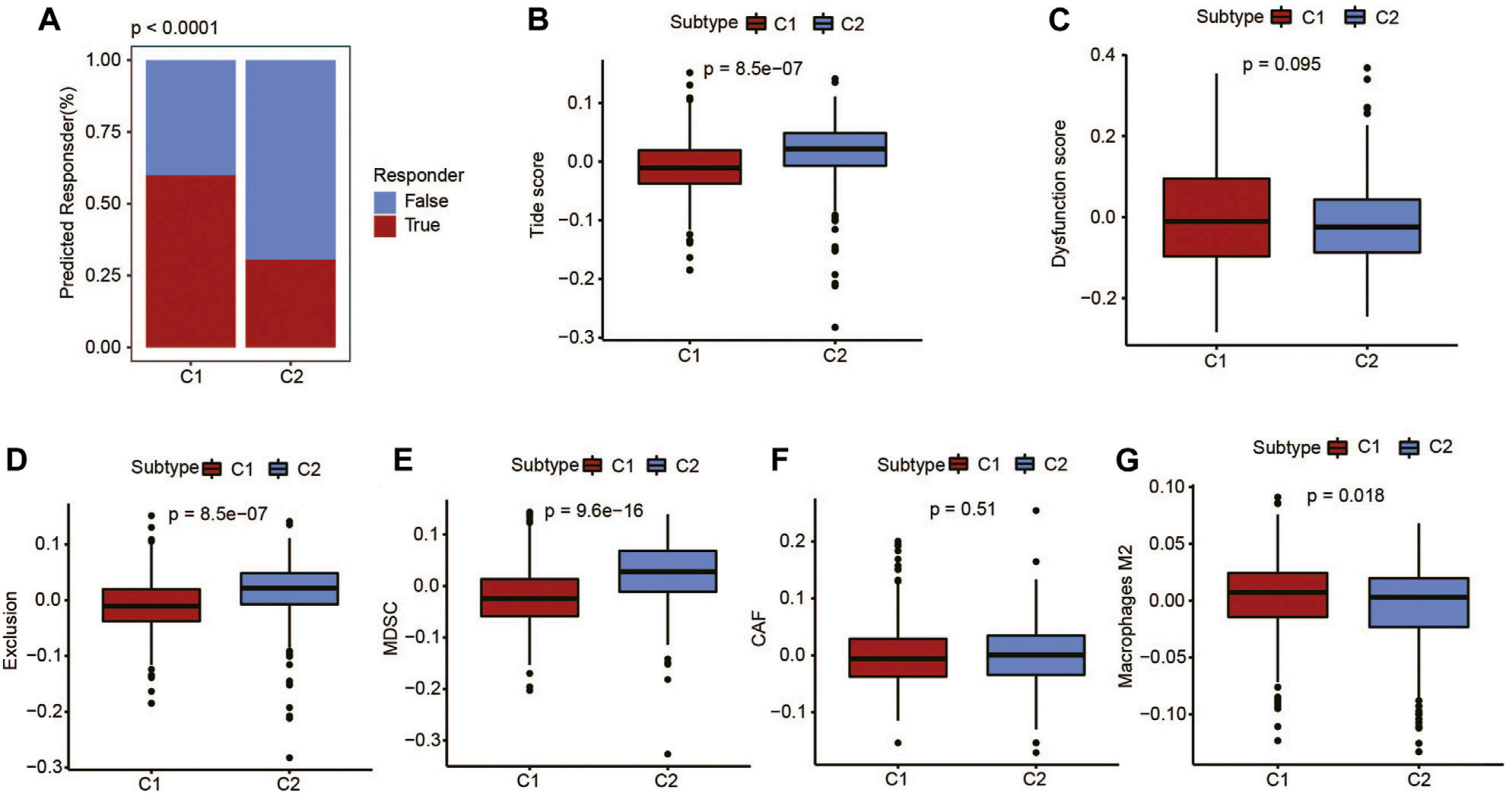
Immunotherapy prediction of UCEC patients using the TIDE algorithm. **(A)** Difference in response to immunotherapy among different metabolic subtypes. **(B)** TIDE score, **(C)** dysfunction score, **(D)** exclusion score, **(E)** myeloid-derived suppressor cell (MDSC) score, **(F)** cancer-associated fibroblast (CAF) score, and **(G)** M2-macrophages score.

In order to model the two primary mechanisms of tumor immune infiltration, TIDE algorithm was applied: the stimulation of T-cell dysfunction accompanying high cytotoxic T-lymphocyte (CTL) infiltration and the prevention of T-cell infiltration with low CTL levels, which estimates the potential response to immunotherapy (Li et al., 2021). Using the TIDE algorithm, the UCEC metabolic C1 subtype was predicted to be more responsive to immunotherapy than the C2 subtype (Figure 4A). Furthermore, based on the dysfunction score and macrophage M2 score, C1 subtype showed a high degree of T-cell dysfunction (Figures 4C, G). C2 subtype included the TIDE score, exclusion score, myeloid-derived suppressor cells, and cancer-associated fibroblasts, indicating a higher degree than the C1 subtype (Figures 4B, D–F). These results showed more robust immune escape characteristics in the C2 subtype compared with the C1 subtype.

**FIGURE 5:**
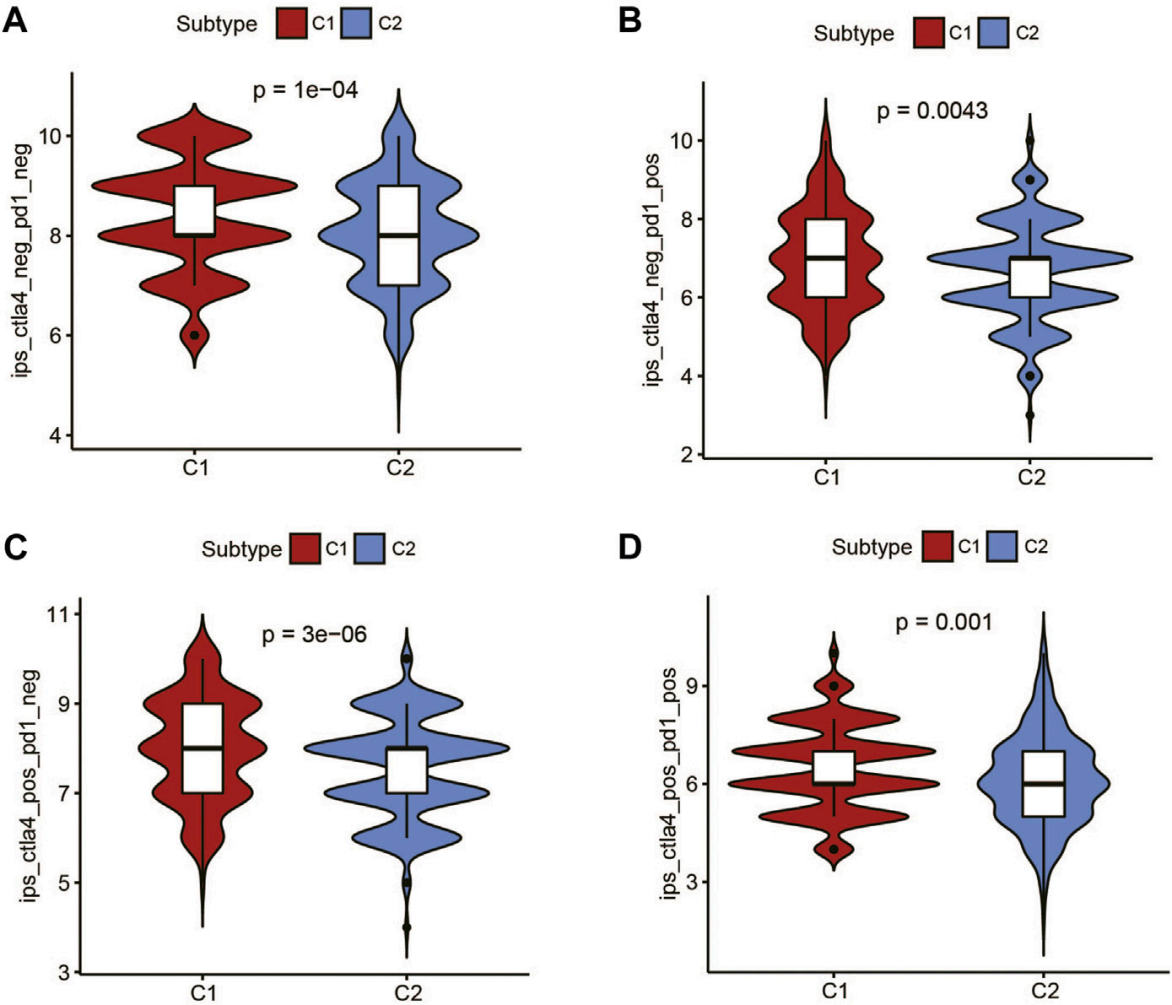
The association between IPS and the metabolic subtypes of UCEC patients. **(A)** IPS of ctla4_neg_pd1_neg in C1 and C2 subtypes. **(B)** IPS of ctla4_neg_pd1_pos in C1 and C2 subtypes. **(C)** IPS of ctla4_pos_pd1_neg in C1 and C2 subtypes. **(D)** IPS of ctla4_pos_pd1_pos in C1 and C2 subtypes.

Recent studies have shown that IPS can predict the therapeutic effects of immune checkpoint inhibitors (ICIs) in cancer patients. This was based on the existing high immunogenic potential. We applied the immunophenotypic score to compare the C1 subtype and the C2 subtype after applying different ICIs (Figures 5A–D). As shown in the figure, regardless of whether cytotoxic T-lymphocyte antigen 4 (CTLA-4) or programmed cell death protein 1 (PD-1) was used for treatment, the immunophenotypic score of the C1 subtype was higher than that of the C2 subtype. This finding indicated that treatment with ICIs was more effective for patients in the C1 subtype.

### Landscape of somatic mutations and copy number alterations of the metabolic subtypes of UCEC

**FIGURE 6:**
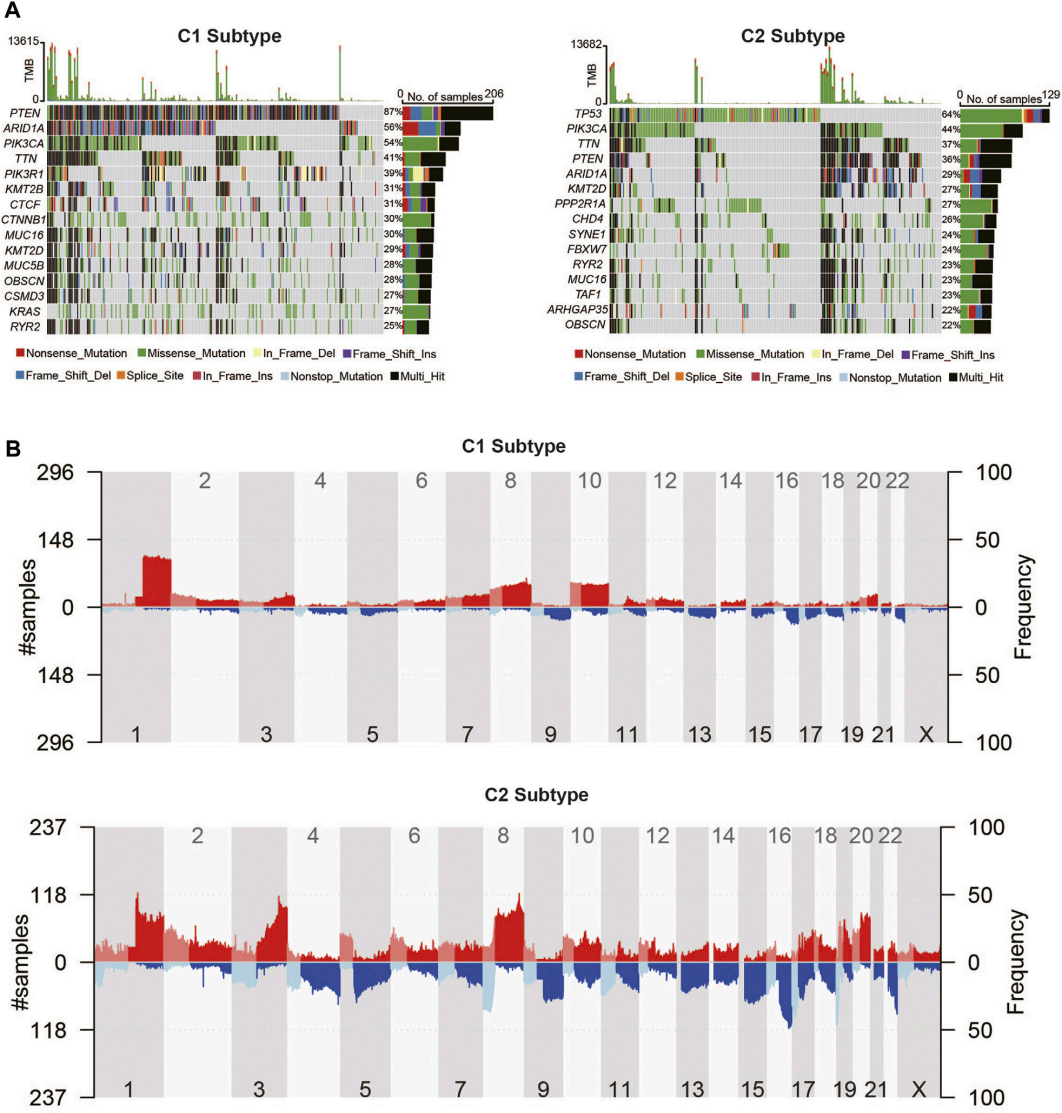
Landscape of somatic mutation and copy number alteration of UCEC metabolic subtypes. **(A)** Somatic mutation waterfall plots of UCEC metabolic C1 and C2 subtypes. **(B)** The distribution of copy number variations of the two metabolic subtypes in human chromosomes.

In order to reveal the genomic difference alterations among the two metabolic subtypes of UCEC patients, we analyzed the top 15 frequency mutation genes in patients of each subtype, which are displayed as a waterfall plot in Figure 6A. ARID1A, PIK3CA, TTN, MUC16, KMT2D, OBSCN, PTEN, and RYR2 were in the top 15 genes of all genes in the two metabolic subtypes. Among them, PTEN contributed 87% to the mutation frequency in the metabolic C1 subtype compared to 36% in the metabolic C2 subtype. The mutation frequency of ARID1A was also higher in C1 (56%) than in C2 (29%). PIK3CA and MUC16 were increased in C1 (54% and 30%) compared with C2 (44% and 23%). The mutation frequencies of TTN, KMT2D, OBSCN, and RYR2 were not significantly different among C1and C2. We found that TP53 had a 64% higher mutation frequency in C2 while it has no mutation in C1, which indicated that the TP53 mutation might play an important role in the metabolic C2 subtype. Then, we compared the CNV alteration in C1 with C2 subtypes, which showed some differences in chromosomal aberrations (Figure 6B). Specifically, the CNV alteration in the C1 and C2 subtypes had several similar amplification or deletion sites. By contrast, the C1 subtype seems to have more amplification region alterations than the C2 subtype. Meanwhile, the C2 subtype was revealed to have more deletion regions than the C1 subtype. GISTIC2 showed that 3q26.2 was a highly significant amplification in the C1 subtype, which contains the MECOM gene (Supplementary Tables S3–S6). 10q23.31 was a highly significant deletion in the C1 subtype, which contains PTEN and KLLN genes (Supplementary Tables S3–S6). Other amplification and deletion regions in the C1 subtype are shown in Supplementary Figure S6 and Supplementary Tables S3–S6. CCNE1amplification in 19q12 was significantly enriched in the C2 subtype. 19p13.3 was a highly significant deletion in the C2 subtype, which contains DAPK3, EEF2, SNORD37, and so on. Other amplification and deletion regions in the C2 subtype are shown in Supplementary Figure S6 and Supplementary Tables S3–S6.

Furthermore, we observed mutations in 2,752 metabolic genes. As shown in Supplementary Figure S7, the top two most frequently mutated metabolic genes in the C1 and C2 subtypes were the same, but the mutation frequency was not the same. PTEN contributed 87% to the mutation frequency in the metabolic C1 subtype compared to 36% in the metabolic C2 subtype. Also, PIKCA is more frequently mutated in the C1 subtype compared with the C2 subtype.

### Prognostic risk model based on characteristic genes of the metabolic subtypes of UCEC

**FIGURE 7:**
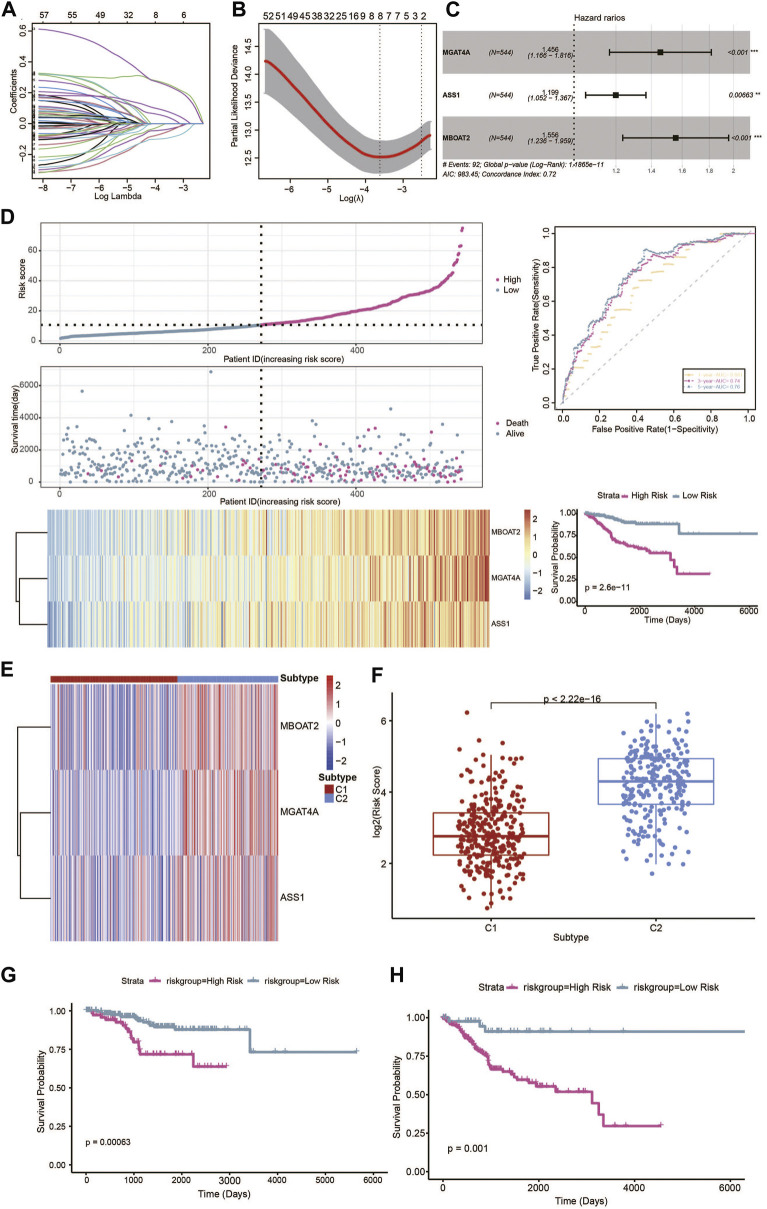
Prognostic risk model based on metabolic characteristic genes of the metabolic subtypes in UCEC. **(A)** LASSO coefficients of the metabolic genes. **(B)** Cross-validation of gene selection using 1-SE criteria in the LASSO regression analysis. **(C)** The forest plot of multivariate Cox regression analysis. **(D)** The predictive value of gene signature in the training dataset. **(E)** Heatmap of the expression levels of signature genes in the metabolic subtypes. **(F)** Distribution of risk scores in the UCEC metabolic subtypes. **(G)** Survival analysis of the metabolic-related signature in the UCEC metabolic C1 subtype. **(H)** Survival analysis of the metabolic-related signature in the UCEC metabolic C1 subtype.

Among the 326 different characteristic DEGs of the metabolic subtypes based on univariate Cox analysis, 183 genes were shown to be significantly correlated with the prognosis of patients, which was confirmed in the LASSO regression analysis. We identified six genes using LASSO-penalized Cox regression analysis in the training set (Figures 7A, B). Subsequently, multivariate Cox regression analysis was used to establish the metabolic signature, and three genes were finally selected as predictors of OS in UCEC patients (Figure 7C). UCEC samples were divided into high-risk and low-risk groups according to the median expression level of the risk score, and the Kaplan–Meier analysis showed significant differences in survival between the groups in the training sets. To further explore the prognostic accuracy of our signature, we performed ROC analysis, with areas under the curve >.69 for 1-, 3-, and 5-year OS times (Figure 7D). Furthermore, the expression profile of the metabolic signature genes was distinct in the two metabolic subtypes (Figure 7E). The C2 subtype has a higher score than the C1 subtype (Figure 7F). Survival analysis revealed that higher scores exhibited significantly poorer prognosis of patients in each UCEC metabolic subtype (Figures 7G, H). The results are consentient with the aforementioned data that the C2 subtype had the worst prognosis.

### Validation of the risk model in TCGA validation set

**FIGURE 8:**
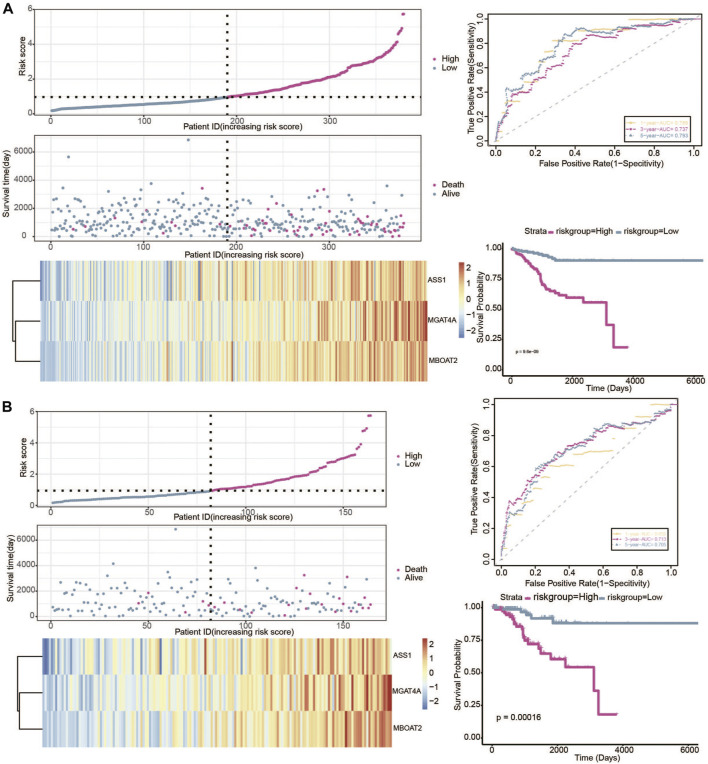
Verification of the predictive value of gene signature in the 2 validation datasets. **(A)** Risk plot, ROC curve, and KM curve of high-risk and low-risk groups in the validation1 set. **(B)** Risk plot, ROC curve, and KM curve of high-risk and low-risk groups in the validation2 set.

In our study, the TCGA patients were randomly divided into 7:3 (380 samples:164 samples) and datasets were selected as validation sets, namely validation1 set and validation2 set. In the two validation sets, patients were also divided into a high-risk group and low-risk group. The KM plot showed significant statistical differences in the survival probability of the high- and low-risk groups, and the high-risk group had a lower survival probability. ROC curves also further reflected the stable sensitivity and specificity of the prognostic model (Figures 8A, B).

### Gene expression verification and genetic alteration analysis in the UCEC metabolic risk model

**FIGURE 9:**
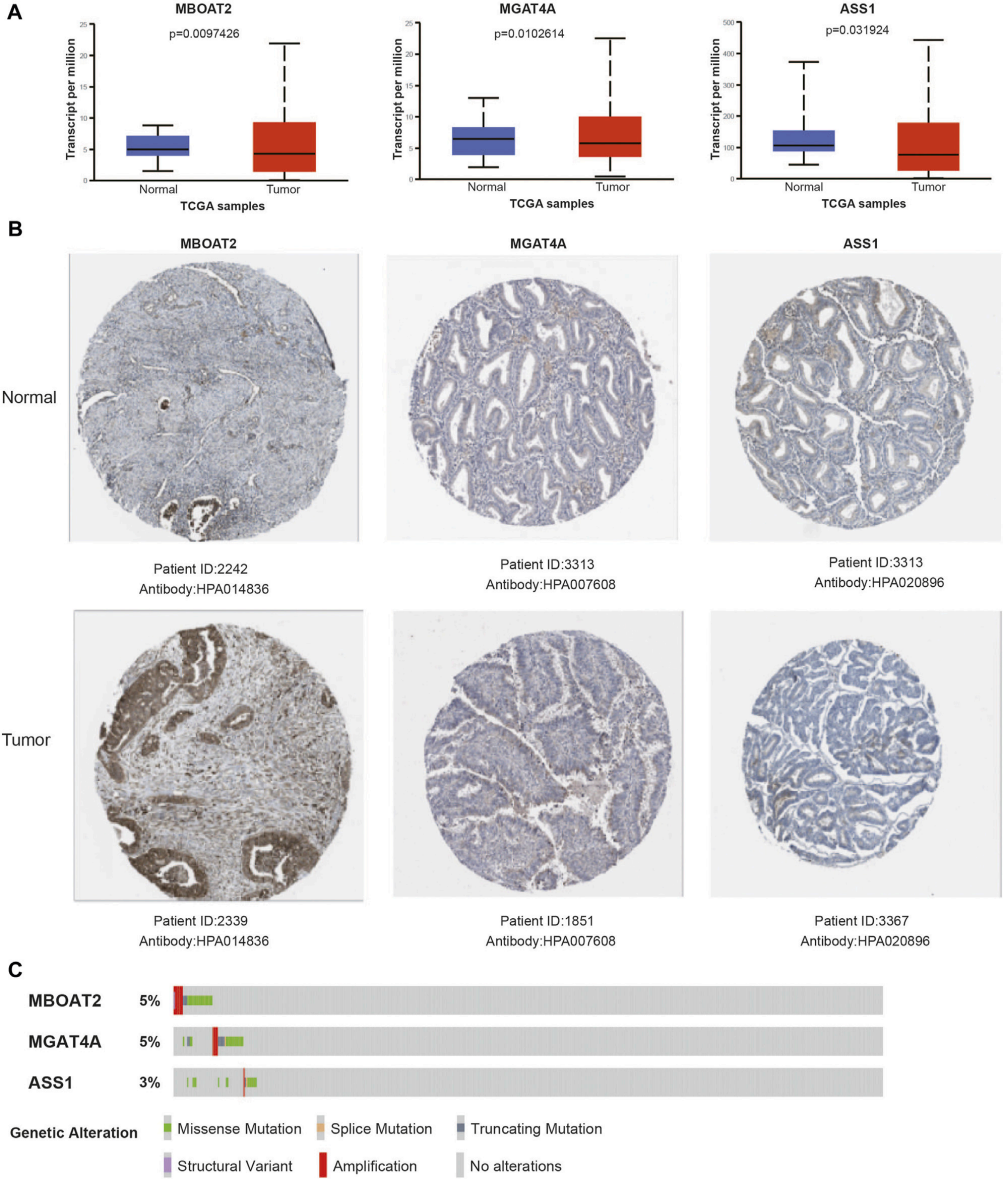
Verification of gene mRNA and protein expression in the risk model. **(A)** The mRNA expression levels of signature genes in UCEC tumor and normal samples. **(B)** Representative protein expression levels of each gene in tumor and normal tissues. **(C)** Genetic alteration oncoplot of the three genes in the UCEC risk model.

In order to verify the expression of the three genes in the risk model, the UALCAN database was used to visualize their mRNA expression levels and found MBOAT2 and MGAT4A were upregulated in tumors, while ASS1 was downregulated (Figure 9A). Similarly, immunohistochemical results of these three genes from The Human Protein Atlas have similar trends in protein expression levels (Figure 9B). The cBioPortal online tool was utilized for genetic alteration analysis, and oncoplot showed that the frequency of these three genes was approximately 5% in the TCGA UCEC cohort (Figure 9C).

## Discussion

According to the traditional classification (based on some features, e.g., different origins, pathogeneses, and genetic characteristics), UCEC can be separated into type I and type II subtypes (Bokhman, 1983). Type I UCEC is estrogen-dependent and has a good prognosis (Hussein and Soslow, 2018). On the other hand, Type II UCEC is non-estrogenic and has a poor prognosis (Carlson and Nastic, 2019). In recent years, UCEC increased with a younger trend, with incidence and mortality increasing. Early-stage UCEC could be surgically removed followed by chemoradiotherapy, with a 5-year survival rate of up to 90% (Guo et al., 2022). Metabolism has been considered one of the key characteristic features of cancer. In this study, based on the metabolic expression profiles, we divided the UCEC patients into two metabolic subtypes and found that the two metabolic subtypes showed distinct differences in many features, such as patient survival outcomes, metabolic signatures, immune signatures, genomic signatures, and immunotherapy efficiency.

In detail, the results showed that the C1 subtype had more active metabolic pathways compared with the C2 subtype. Therefore, we defined the C1 subtype as an active metabolic subtype and the C2 subtype as a metabolic exhausted subtype. We all know the immune cell microenvironment and the PI3K-Akt, Wnt, and MARK signaling pathways were involved in UCEC development (Zhou et al., 2020). Similarly, cancer pathway signatures such as the PI3K-pathway and Wnt-pathway were also enriched in the C1 subtype, leading to tumor development, which is consistent with the Type I UCEC report in other studies (Hussein and Soslow, 2018). Moreover, tumor immune microenvironment analysis demonstrated that the C1 subtype had the higher immune score, stromal score, and ESTIMATEScore. These data suggested that C1 subtype may have a high heterogeneity. Despite these findings, some other studies have been conducted on UCEC thus far. Based on the WNT metabolic gene family, UCEC was classified into two subtypes using real-world data (Hu et al., 2021).

We compared the mutation and cnv alteration between the C1 subtype and C2 subtype in further analysis. The results showed that the C1 subtype has a higher mutation in genes such as PTEN, PIK3R1, KRAS, ARID1, and CTNNB1, which has a good prognosis. Interestingly, the C2 subtype had a higher mutation in TP53, FBXW7, and PPP2R1A genes, which had a poor prognosis. Also, the C2 subtype seems to have a higher CNV alteration than C1. These results have higher similarity with previous studies (Carlson and Nastic, 2019; Hussein and Soslow, 2018; Kandoth et al., 2013). In previous analysis studies (Carlson and Nastic, 2019; Hussein and Soslow, 2018; Kandoth et al., 2013), type I UCEC is associated with mutations, such as PTEN, KRAS, ARID1A, PIK3CA, and CTNNB1 and microsatellite instability (MSI). P53 mutations and HER2 overexpression characterize type II UCEC. Also, the C1 subtype and the C2 subtype in this study have similar molecular features to the TCGA POLE hyper-mutation subtype and high-copy number type (such as the p53 gene mutation).

Then, we provide new insight into the treatment response relationship between the metabolic classifications of UCEC. Regarding immunotherapy response prediction, the C1 subtype has a higher immune infiltration state, which is typically associated with a good prognosis. TIDE is a new computing architecture that predicts the immunotherapy response mechanisms. The results showed that the C1 subtype was predicted to be more responsive to immunotherapy. Together, these findings explain that the C1 subtype has high immune infiltration and a good prognosis. Using TCGA data, UCEC was identified in three immune subtypes, with different tumor purities, immune scores, stromal scores, fractions of different immune cells among UCEC subtypes (Liu et al., 2021). All these results suggest that the tumor immune microenvironment has different landscapes in UECE patients and subtypes.

It is well known that there is already a well-established UCEC molecular classification by previous studies using TCGA (Kandoth et al., 2013), which classified endometrial cancer into four subtypes: POLE ultra-mutated, microsatellite instability hyper-mutated, copy-number low, and copy-number high. In this study, we identified two metabolic subtypes based on 2,752 metabolic genes. Comparing these two metabolic genes with TCGA’s four subtypes, we found some similarities, such as PTEN being highly mutated in the C1 subtype, which is consentient with TCGA POLE ultra-mutated subtype. Meanwhile, we found the C2 subtype has a higher CNV alteration and frequent TP53 mutations (64%), which was also found in TCGA copy-number high. Furthermore, we also have some new discoveries, like the C1 subtype predicted to be more responsive to immunotherapy, also with more mutations. These could provide evidence for the treatment of endometrial cancer.

Finally, at the end of this study, we developed a metabolism-related model signature, which had a better performance for prognosis prediction in UCEC. The model signature consisted of 4 metabolic genes, which were differentially expressed between the metabolic subtypes of UCEC but were also significantly related to the patient’s prognosis. Patients with high risk-scores showed significantly poor prognosis in both training and validation datasets.

## Conclusions

Using public TCGA cohort data, we accessed a molecular classification of UCEC patients based on metabolism-related subtypes. Then, we comprehensively described the subtypes’ metabolic characteristics, prognostic characteristics, immune infiltration, genetic alteration, and responses to immunotherapy. However, some flaws are also present in this study. First, a larger sample size and further basic experiments are needed to support our metabolic subtype. Then, validation of the classification in clinical samples is also imperative. Overall, our works provide important information for personalized therapies and prognostic predictions.

## Data Availability

The detailed information of UCEC samples was downloaded from The Cancer Genome Atlas (TCGA) (https://portal.gdc.cancer.gov) and the UCSC Xena browser (https://gdc.xenahubs.net). The 2,752 metabolism-related genes’ list was acquired from a previously published article. Immune infiltration was acquired from CIBERSOT databases (https://cibersort.stanford.edu/). Tumor Immune Dysfunction and Exclusion (TIDE) was applied for immune checkpoint blockade therapy. The IPS of UCEC was downloaded from the TCIA database (https://tcia.at/).

## Abbreviations

UCEC, uterine corpus endometrial carcinoma; TCGA, The Cancer Genome Atlas; DEGs, differentially expressed genes; CNV, copy number variation; OS, overall survival; HCC, hepatocellular carcinoma; NCCN, National Committee on Computer Network; MSI, microsatellite instability; NSMP, non-specific molecular variation; LASSO, least absolute shrinkage and selection operator; GO, Gene Ontology; KEGG, Kyoto Encyclopedia of Genes and Genomes; GSVA, Gene Set Variation Analysis; ROC, receiver operating curve; AUC, area under the curve; ESTIMATE, Estimation of Stromal and Immune cells in Malignant Tumor tissues using Expression; MAF, Mutation Annotation Format; FDR, False Discovery Rate; FC, fold change; TIDE, tumor immune dysfunction and exclusion; ICB, immune checkpoint blockade; IPS, immune-phenotyping score.
