# Supplementary figures and images for: Metabolism-associated molecular classification of uterine corpus endometrial carcinoma
Source: Front Genet. 2023 Jan 16;14:955466. doi: 10.3389/fgene.2023.955466 (PMC9885131; doi:10.3389/fgene.2023.955466)

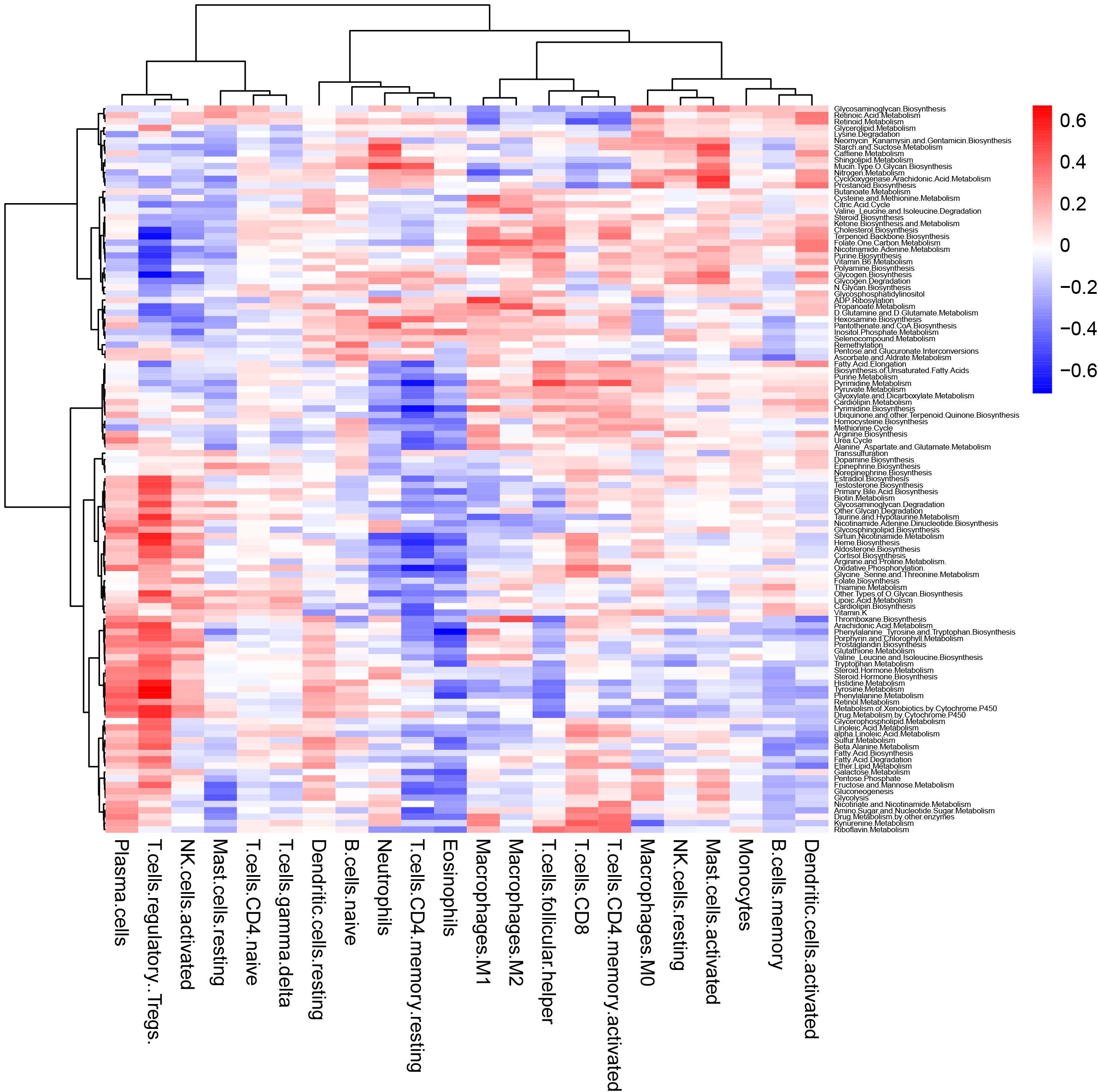

Supplement: Supplementary file 1 [file Image3.JPEG]

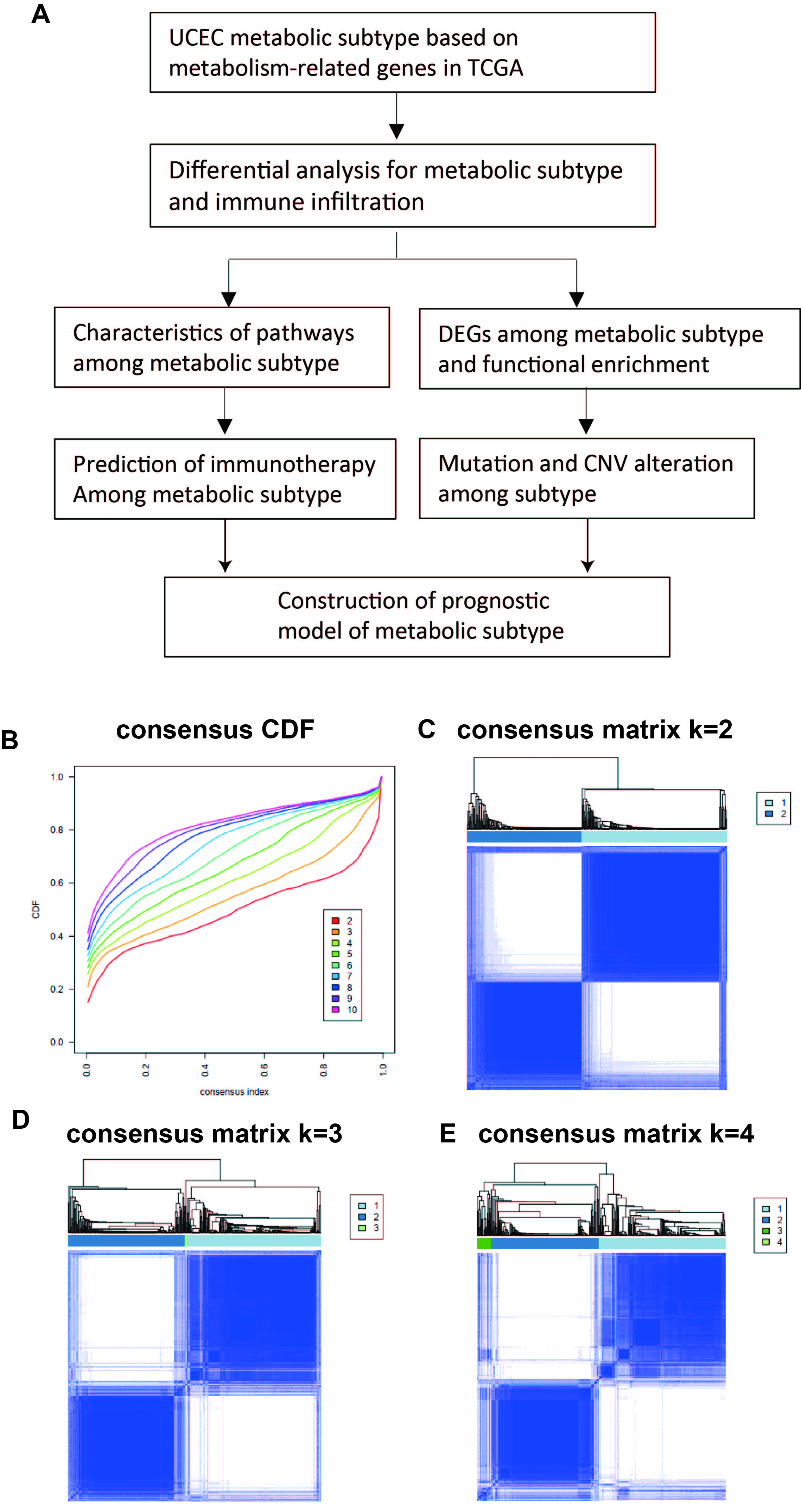

Supplement: Supplementary file 2 [file Image1.JPEG]

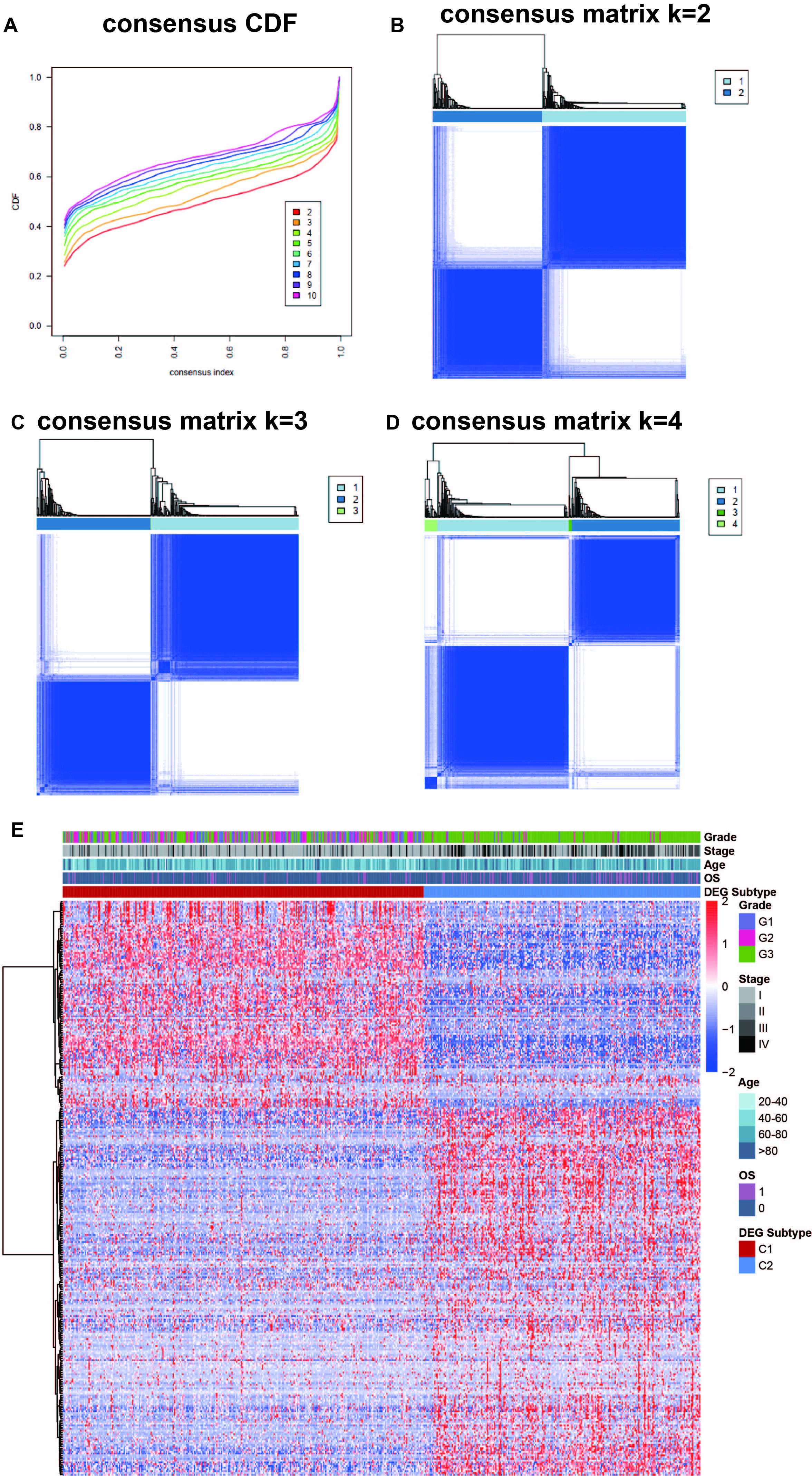

Supplement: Supplementary file 3 [file Image4.JPEG]

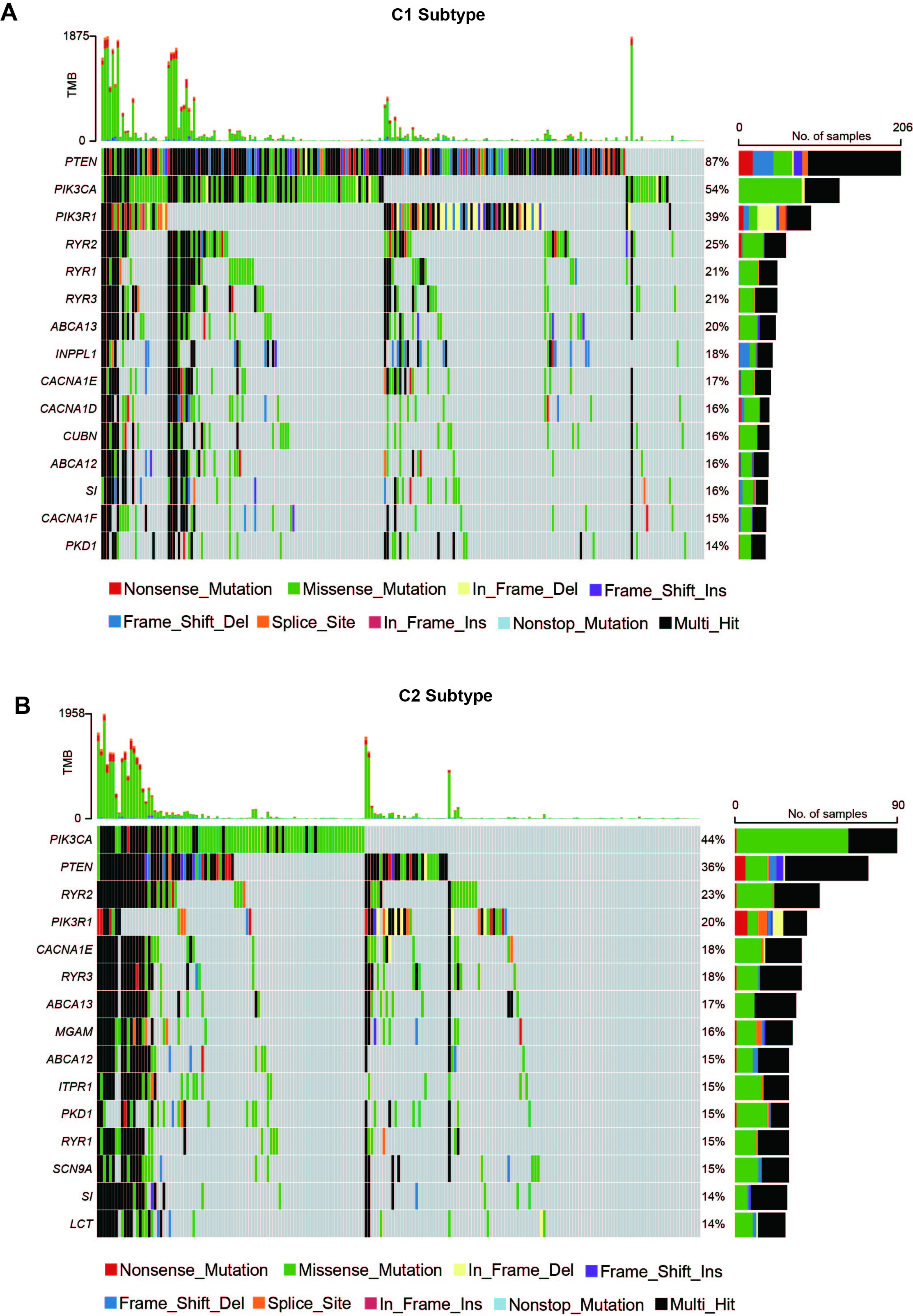

Supplement: Supplementary file 4 [file Image7.JPEG]

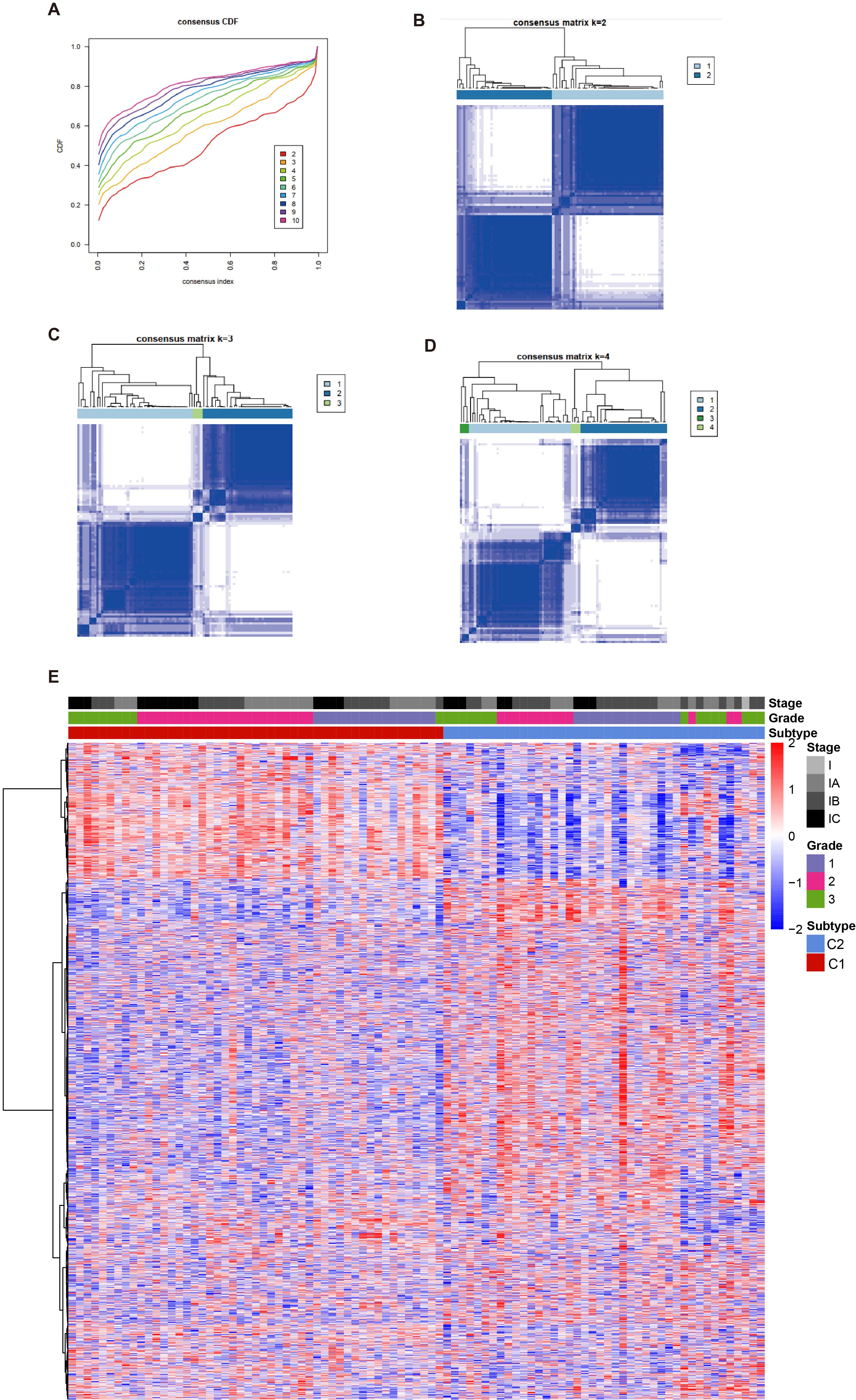

Supplement: Supplementary file 5 [file Image2.JPEG]

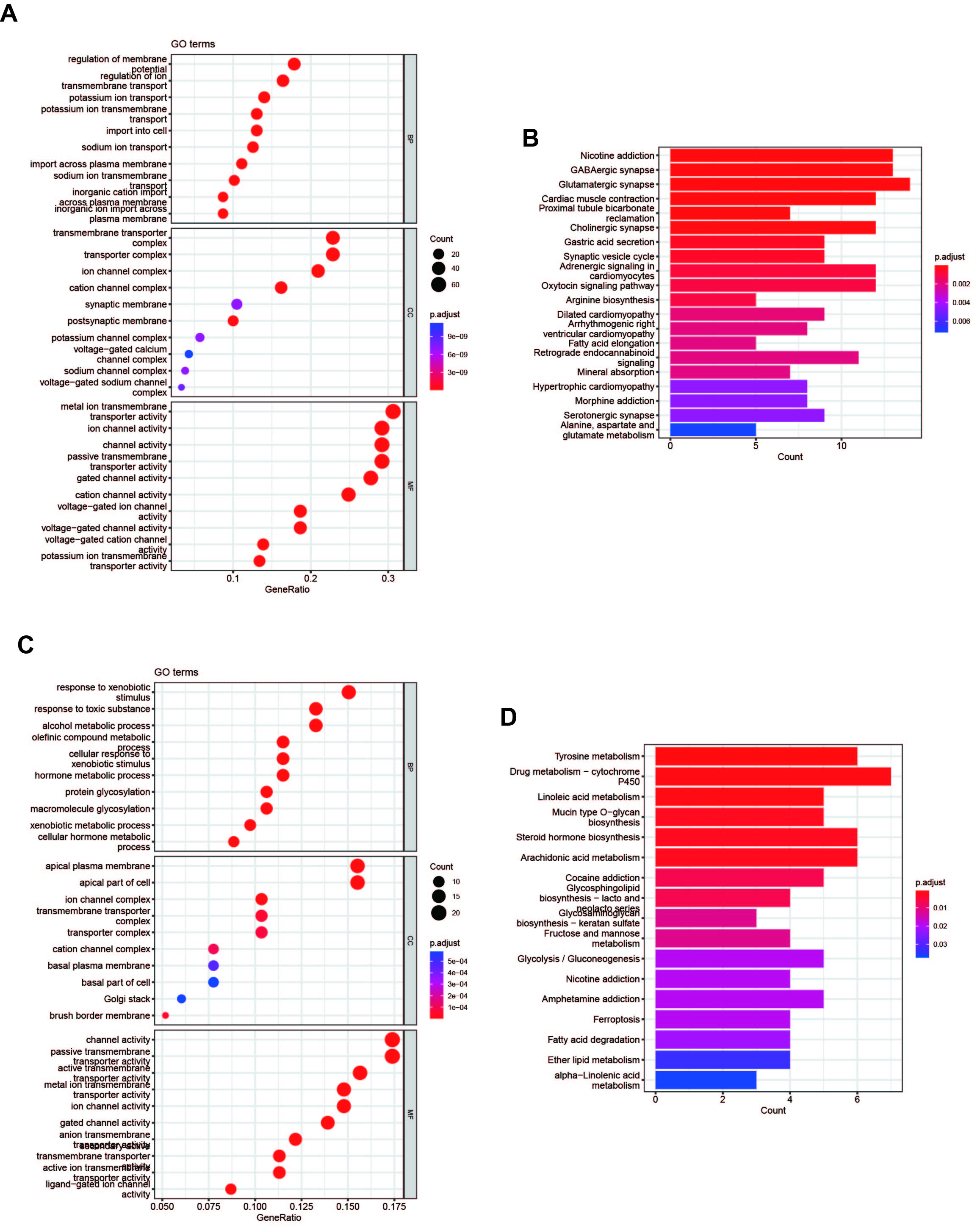

Supplement: Supplementary file 6 [file Image5.JPEG]

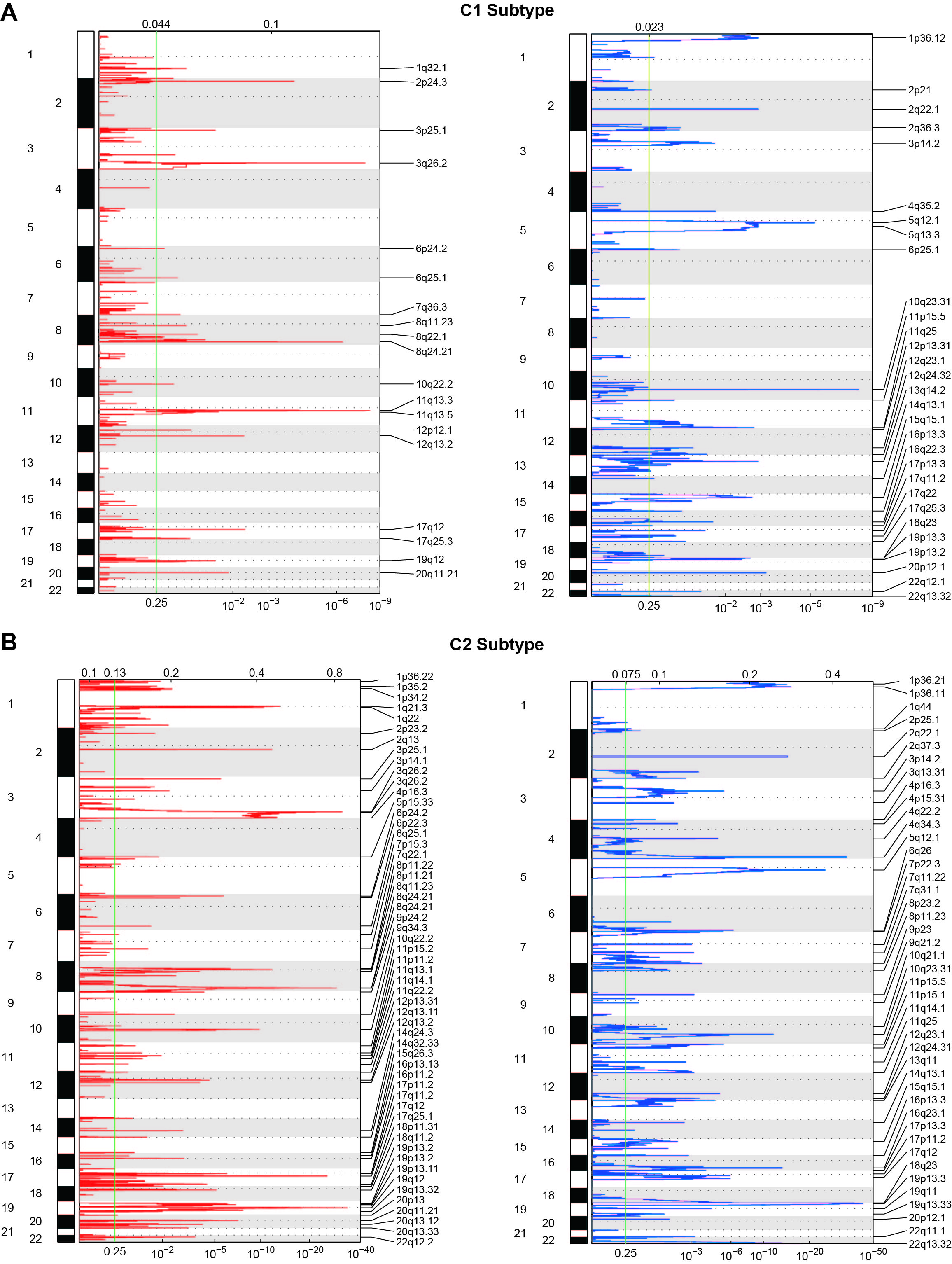

Supplement: Supplementary file 8 [file Image6.JPEG]
